# Genetic Profiles and Antimicrobial Resistance Patterns of *Salmonella* Infantis Strains Isolated in Italy in the Food Chain of Broiler Meat Production

**DOI:** 10.3390/antibiotics9110814

**Published:** 2020-11-16

**Authors:** Patrizia Casagrande Proietti, Valentina Stefanetti, Laura Musa, Alessia Zicavo, Anna Maria Dionisi, Sara Bellucci, Agnese La Mensa, Laura Menchetti, Raffaella Branciari, Roberta Ortenzi, Maria Pia Franciosini

**Affiliations:** 1 Department of Veterinary Medicine, Via S. Costanzo 4, 06126 Perugia, Italy; valentina.stefanetti@unipg.it (V.S.); laura.musa@studenti.unipg.it (L.M.); sara.bellucci@studenti.unipg.it (S.B.); agnese.lamensa@you.unipa.it (A.L.M.); raffaella.branciari@unipg.it (R.B.); maria.franciosini@unipg.it (M.P.F.); 2 Istituto Zooprofilattico Sperimentale dell’Umbria e delle Marche “Togo Rosati”, Via G. Salvemini, 1, 06126 Perugia, Italy; a.zicavo@izsum.it (A.Z.); r.ortenzi@izsum.it (R.O.); 3 Istituto Superiore di Sanità, Viale Regina Elena, 299, 00161 Roma, Italy; annamaria.dionisi@iss.it; 4 Department of Agricultural and Food Sciences, Viale Fanin 42, 40127 Bologna, Italy; laura.menchetti7@gmail.com

**Keywords:** *Salmonella* Infantis, antimicrobial susceptibility, ESBL, genetic profile, PFGE

## Abstract

This work aimed to evaluate the antimicrobial susceptibility of 87 *Salmonella* Infantis strains isolated in Italy from 2016 to 2019 along the food chain of broiler meat production and in humans and to determine the genetic profiles of the strains in order to establish a possible correlation with the antimicrobial pattern. All isolates were tested by the disk diffusion method to evaluate antimicrobial susceptibility toward sixteen antimicrobials, and the broth microdilution method was used to confirm extended spectrum β-lactamase (ESBL) production. PCR and pulsed field gel electrophoresis (PFGE) were applied to characterize ESBL-encoding and AmpC β-lactamase genes and to analyze the *S*. Infantis strains genetic profiles respectively. *S*. Infantis isolates showed high prevalence of resistance, in particular toward nalidixic acid (97.7%), tetracycline (96.5%), sulphamethoxazole/trimethoprim (91%) and cefepime (72.4%). The 80.5% of isolates were ESBL, cefotaxime-resistant, carrying the bla_CTX-M1_ gene. The most prevalent PFGE profile was XbaI.0126 (35.6%). The remaining strains had a genetic homology from 81% to 97% with the XbaI.0126 profile. The strains belonging to these profiles were isolated from different matrices collected along the broiler food chain independently on the year and from the region and there was no correlation between the PFGE profiles and resistance patterns. We found two ESBL-producing *S.* Infantis strains with the same XbaI.2621 profile isolated from humans and from poultry feces, not yet reported in Italy. Our findings confirmed the diffusion of ESBL-multi drug resistant (MDR) *S*. Infantis along the broiler food chain and in humans and underlined the importance of continuous monitoring to control and to reduce the prevalence of this bacterium, applying a global One Health approach.

## 1. Introduction

*Salmonella enterica* subsp. *enterica* serovar Infantis (*S.* Infantis) has emerged as the fourth most frequent serovar causing human salmonellosis in Europe from 2006 to 2016 [1], becoming a serious public health concern. Although this serovar is non-host adapted it has been isolated from a wide range of animals, and broilers and broiler meat are considered one of the principal sources of human infection [1,2]. According to the last European Food Safety Authority (EFSA) and European Centre for Disease Prevention and Control (ECDC) report, *S*. Infantis was the commonest reported serovar from broiler flocks (46.5%) and broiler meat (50.6%) of all serotyped *Salmonella* isolates reported from these sources [3]. In Italy, *S*. Infantis isolates has been reported in humans (39.4%) from food and animal sources and data obtained from cross-sectional studies in broilers at slaughter revealed that *S*. Infantis accounted for 75% and 90% of all isolates detected, respectively in 2014 and 2016 [4,5]. The surveillance and monitoring of *S*. Infantis foodborne outbreaks appear crucial both at the national and international level. Although new-generation molecular techniques have become an essential support for this purpose, the application of pulsed field gel electrophoresis (PFGE) still remains a powerful tool for establishing genetic relatedness of different *S.* Infantis strains [6,7].

Over recent years, the increasing incidence of *S.* Infantis infection in humans and animals has also been complicated by the spread of multidrug resistant (MDR) clones in several European countries, including Switzerland [8], Slovenia [9], Hungary, Austria, Poland [10], Israel [7], Germany [11] and Italy [12,13]. In human medicine, MDR *Salmonella* strains have been linked with longer duration of hospitalization, prolonged illness and increased mortality rates compared to drug-susceptible strains [14]. The European clones of *S*. Infantis of broiler origin are mainly characterized by a pattern of resistance to nalidixic acid, sulphamethoxazole, streptomycin and tetracycline (NaSSuT) [10]. The recent emergence among people and broilers of *S*. Infantis strains with extended spectrum β-lactamase (ESBL)-mediated resistance to third-generation cephalosporins [8,12], with reduced susceptibility to fluoroquinolones [15] is particularly interesting.

This poses serious public health implications, especially because these two classes are critically important human antimicrobials since they are the drug of choice for human salmonellosis [16].

There is evidence that the acquisition of a novel megaplasmid confers resistance to multiple drugs in *S*. Infantis isolates [17]. It has been demonstrated that Italian *S*. Infantis strains isolated from chickens, chicken meat and humans harbored the pESI-like megaplasmid, similar to that described in Israel in 2014, which carries the ESBL gene *bla_CTX-M-1_* and further genes conferring antibiotic-resistance [12]. Additionally, it has recently been demonstrated in *S*. Infantis that colistin resistance, mediated by the *mcr-1* gene, is also carried by another conjugative plasmid [18]. This point also represents a serious public health concern, since the antimicrobial resistance genes can be easily transferred from poultry into the human food chain.

The aims of the present study were to evaluate the *S.* Infantis antimicrobial susceptibility pattern, and the prevalence of *S.* Infantis ESBL isolated from the broiler food chain and humans in Italy. The genetic profiles of strains were also investigated by PFGE in order to determine a possible correlation with the antimicrobial pattern.

## 2. Results

### 2.1. Antimicrobial Susceptibility and Characterization of ESBL-Encoding and AmpC β-Lactamase Genes

The results of antimicrobial susceptibility tests are shown in Table 1. In particular, 85 isolates (97.7%) showed resistance toward nalidixic acid, 84 strains (96.5%) were resistant to tetracycline and 79 strains (91%) were resistant to sulphamethoxazole/trimethoprim. Seventy isolates (80.5%) displayed resistance toward cefotaxime. Sixty-three (72.4%) and 12 (13.8%) isolates were resistant and intermediate to cefepime, respectively. The 56.3% and 18% of isolates were intermediate and resistant to ciprofloxacin, respectively. Eighteen (20.7%) and 14 (16%) isolates were resistant to chloramphenicol and gentamicin, respectively. With regard to carbapenems, 9 strains (10.3%) were intermediate to imipenem and 5 (5.7%) and 10 (11.5%) isolates were resistant and intermediate to meropenem, respectively.

As indicated in Table 2, a total of 12 multi-resistance patterns were observed. In particular, 85 strains (97.7%) showed a multidrug resistant phenotype being resistant to 3, 4, 5 or 6 classes of antimicrobials. The highest prevalence of resistance was found toward beta/quin/sulph/tetra (*n* = 45, 51.7%; chi-square goodness of fit: *p* < 0.001), followed by beta/quin/clor/sulph/tetra (*n* = 8, 9.2%), quin/sulph/tetra (*n* = 6, 6.9%), and beta/amino/quin/sulph/tetra (*n* = 6, 6.9%).

**Figure 1:**
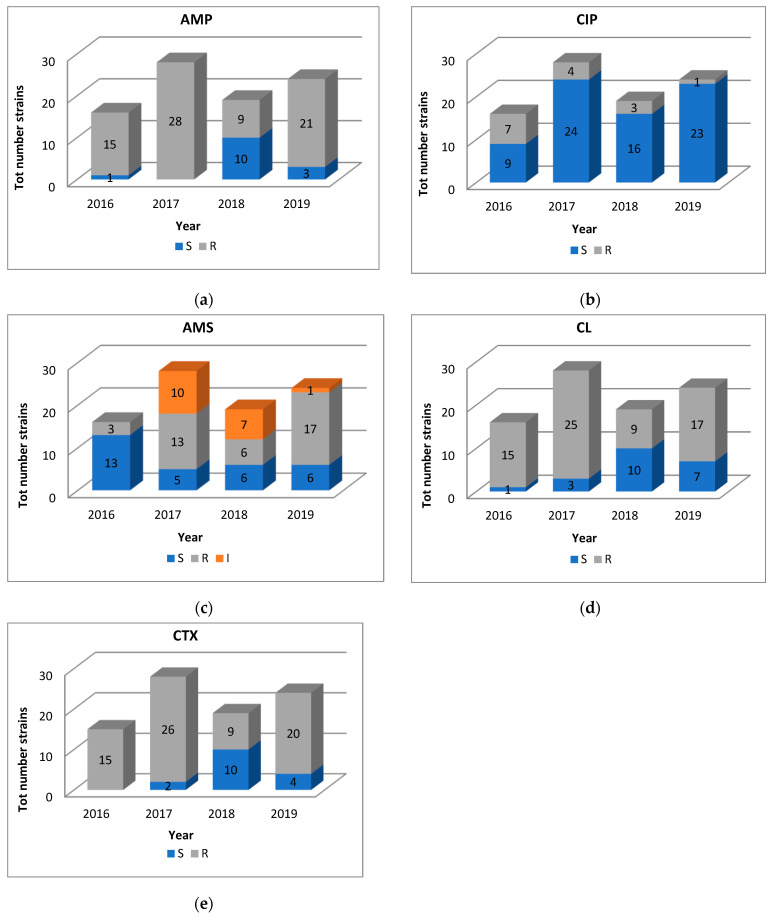
Number of *S*. Infantis isolates resistant, intermediate and susceptible to ampicillin (AMP, Panel (**a**)), ciprofloxacin (CIP Panel (**b**)), ampicillin/sulbactam (AMS, Panel (**c**)), cephalexin (CL, Panel (**d**)) and cefotaxime (CTX, Panel (**e**)) over the four-year study period.

Only isolates resistant to beta/quin/sulph/tetra were analyzed by the binomial logit model as this variable complied with the events per variable (EPV) rule. The odds of beta/quin/sulph/tetra multiresistance reduced over time, from 2016 (62.5%) to 2019 (16.7%; aOR = 0.109, 95%CI = 0.023–0.507, *p* < 0.01). Over the study period, we observed some differences in the S. Infantis strains antimicrobial susceptibility profile, as shown in Table S1. In 2019 we observed a significant reduction in resistance for ciprofloxacin (aOR = 0.052, 95%CI = 0.005–0.501; *p* < 0.05) and a significant increase in resistance for ampicillin/sulbactam (aOR = 9.347, 95%CI = 1.950–44.803; *p* < 0.01) compared to 2016. Additionally, multiple comparisons showed that in 2018 there was a reduction of ESBL-S. Infantis isolates. In fact in this year, S. Infantis strains had the lowest prevalence of resistance for ampicillin (*p* < 0.001), cephalexin (*p* < 0.001) and cefotaxime (*p* < 0.001) (Figure 1)

No difference in antibiotic resistance among matrices was found (Table S2). Seventy strains (80.5%) with the ESBL phenotype contained the bla_CTX-M1_ gene and two out of these isolates were also bla_CMY-2_ positive. Table 2 shows PCR results and the relative antibiotic-resistance pattern, demonstrating that all the 70 ESBL-*S*. Infantis had a multi-drug resistant phenotype. Seventeen *S*. Infantis strains of our study (19.5%) resulted non-ESBL. Twelve out of 17 strains (70.5%) showed resistance to quin/sulph/tetra (Table 3).

### 2.2. Macrorestriction PFGE Cluster Analysis

**Figure 2:**
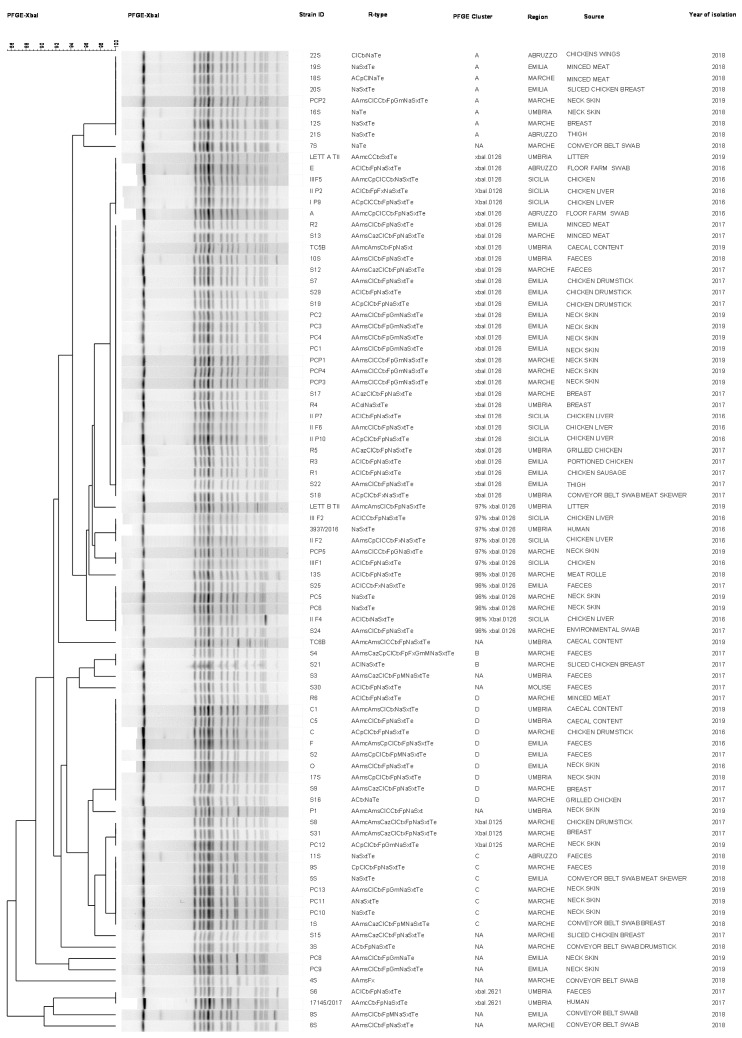
XbaI PFGE macrorestriction cluster analysis and antimicrobial resistance pattern of 87 *S*. Infantis strains. * R-type: ampicillin (A), amoxicillin/clavulanic acid (Amc), ampicillin/sulbactam (Ams), ceftazidime (Caz), ciprofloxacin (Cp), cefalexin (Cl), cloramphenicol (C), cefotaxime (Ctx), cefepime (Fp), cefoxitin (Fox), gentamicin (Gn), imipenem (I), meropenem (M), nalidixic Acid (Na), trimethoprim/sulphametoxazole (Sxt) and tetracycline (Te).

The macrorestriction PFGE cluster analysis is shown in the dendrogram (Figure 2). According to ECDC nomenclature, the dendrogram analysis showed that 31 out of 87 isolates (35.6%; chi square goodness of fit: *p* < 0.001) had the XbaI.0126 profile and the remaining 56 isolates (64.4%) had a genetic homology ranging from 81% to 97% with the XbaI.0126 profile. In particular, 6/56 had 96% homology and 6/56 had 97% with the XbaI.0126 profile. In this latter profile one isolate out of the two human strains was included. Twelve strains belonged to restriction profiles not assigned (NA) yet. Among these 56 strains, there were four profile clusters not yet labeled by ECDS nomenclature, which we named A (*n* = 8), B (*n* = 2), C (*n* = 7) and D (*n* = 10). The strains belonging to these clusters were isolated from different matrices collected along the broiler food chain between 2016 and 2019 in North, Central and South Italy. Three out of 56 strains had an XbaI.0125 profile and they were all isolated in the Marche region (Central Italy) in 2017 and 2019 from broiler meat products. Two out of 56 strains belonged to the XbaI.2621 profile and were isolated in Umbria (Central Italy) in 2017 from human and broiler feces. Twenty-nine out of 31 strains (93.5%) belonging to the Xbal.0126 profile, 5 of 6 strains having a 97% homology with the XbaI.0126 profile, 4 of 6 the strains having a 96% homology with the XbaI.0126 profile, 8 of 10 strains with D profile, 3 strains with the Xbal.0125 profile, 2 strains with the Xbal.2621 profile and 9 of 12 profiles not yet assigned were ESBL producers and showed the same antimicrobial-resistance core (cefotaxime, cefepime, nalidixic acid, trimethoprim/sulphametoxazole and tetracycline). The two strains belonging to cluster B and seven strains with cluster C had the same resistance core to nalidixic acid, trimethoprim/sulphametoxazole and tetracycline. Cluster A had eight strains with a core of resistance to nalidixic acid and tetracycline.

## 3. Discussion

*S*. Infantis is the serovar most frequently isolated in broiler flocks and the fourth most common in breeding flocks and laying hens in the European Union [19]. In this study, we firstly determined the phenotypic resistance patterns of *S.* Infantis isolated from the food chain of broiler meat production (*n* = 85) and from humans (*n* = 2), and then we evaluated the genetic profiles of the isolates and the possible correlations between specific genotypes and antimicrobial susceptibility patterns. Eighty-five (98%) isolates were multiresistant and 70 (80.5%) isolates were ESBL, carrying *bla_CTX-M-1_.* Our investigation demonstrated that there were no statistically significant differences in the susceptibility of *S.* Infantis isolated from different matrices collected along the food chain of broiler meat production. This suggests that *S*. Infantis-resistant strains can spread and persist easily along the entire broiler production chain once it has become established. Moreover, we found the highest prevalence of *S*. Infantis ESBL-producing among the strains isolated in 2016. On this matter it should be highlighted that in recent years most of the large Italian conventional poultry-producing companies have progressively turned to antibiotic-free and organic lines of products. However, in an our previous work [20] we found high prevalence of *E. coli* commensal strains resistant to tetracycline and nalidixic acid in both in antibiotic free and organic farming due likely to the vertical transmission and/or early contamination at hatch. Furthermore, an external environment contamination by resistant bacteria could be considered in an organic farm. Franco et al. [12] demonstrated that in Italy there is a stable population of *S.* Infantis isolated in chickens, chicken meat and humans harboring the p*ESI*-like megaplasmid carrying the ESBL gene *bla_CTX-M-1_,* and additional genes *tet(A), sul1*, *dfrA1* and *dfrA14* mediating cefotaxime, tetracycline, sulphonamide and trimethoprim resistance. Our results showed that ESBL-positive isolates were resistant toward tetracycline, sulphametoxazole/trimethoprim and nalidixic acid, and 12 out of 17 ESBL-negative isolates, confirming the typical pattern of multiresistance of the European *S*. Infantis clone [8,9,21]. The use of these antimicrobials and also of ampicillin, amoxicillin and quinolones, as therapy and prevention in poultry, has favored the selection of resistant bacteria shareable at the human level through food or environmental contamination, and through direct contact with animals [22]. Twenty-three out of 70 ESBL-producing strains were resistant (*n* = 5) and intermediate (*n* = 9) to meropenem and intermediate (*n* = 9) to imipenem. This finding may be due to carbapenemase-producing *Salmonella*. Roschanski et al. [23] speculated the occurrence of a possible coselection process to explain the consistent reoccurrence of highly similar *Salmonella* carbapenemase-producing isolates or plasmids harboring the *bla_VIM-1_* gene in fattening chicken farms. We also observed that 63 out of 87 isolates were also cefepime-resistant, differently from Thakur et al. [24], who found that all *S*. Infantis isolates were cefepime-susceptible. This finding could be relevant because cefepime is fourth-generation cephalosporin and, as well as the other cephalosporines, they are not licensed in the poultry industry. In this sense, it is to take into consideration the action played likely by the outdoor often contaminated with resistant bacteria present in the soil or shed by wild birds or mammals [25,26]. Moreover a recent review on the important role of the environment in alarming increase of carbapenemase-producing bacteria in pigs, cattle, poultry, fish, wild birds and wild mammals in Europe and in non-European countries should be mentioned [27]. Fu et al. [28] demonstrated an increase of cefepime resistance in *S*. Enteritidis and that the *bla_CTX–M–55_* gene mobilized by the ISEcp1-*bla_CTX–M–55_-*ORF477 transposition unit is the main characteristic shared by cefepime-resistant *S.* Enteritidis. This mechanism plays an important role in the transmission among different bacterial species, becoming a serious public health concern. Hindermann et al. [8] found resistance to ciprofloxacin in 4.6% of the *S.* Infantis isolates and showed an increasing prevalence in the study period (2010–2015). In contrast with their finding, in our study we observed a significant reduction in resistance to ciprofloxacin, due to a more cautious use of fluoroquinolones in the last decade in Italy because they are “critically important antimicrobials”(CIAs) and are considered lifesaving in human medicine [29]. Moreover fluoroquinolones have been overused in the metaphylaxis and therapy of chickens causing a selective pressure on the microbial community with the transfer of resistance determinants among bacterial species [30]. To investigate the genetic profile in *S*. Infantis isolates we used PFGE. Although this procedure has been extensively used worldwide by the PulseNet system, it should be underlined that a lack in the nomenclature of *S*. Infantis profiles assigned by the ECDC exists. In most of the studies on *S*. Infantis, the authors did not apply ECDC nomenclature. This fact leads to difficulty in the ability to compare *S.* Infantis strains isolated from other European and non-European states and in speculating about their circulation. In our study, the most prevalent PFGE profile was XbaI.0126 (35.6%). The remaining strains belonged to clonally related groups, having a genetic homology from 81% to 97% with the XbaI.0126 profile, including the XbaI.0125 cluster. The strains belonging to these profiles, described only in humans by Dionisi et al. [31], were isolated from different matrices collected along the broiler food chain independently on the year and on the region. However, it should be further underlined that in the previous investigations on Italian *S*. Infantis strains the ECDC nomenclature has not been used for the PFGE analysis [4,12]. Finally, there was no correlation neither between the PFGE profiles and resistance patterns nor between matrices and pulsotypes. In the present study, we found two *S*. Infantis strains with the same XbaI.2621 profile, isolated from humans and from poultry feces. These profiles, not yet reported in Italy, were ESBL and resistant to cefepime, nalidixic acid, tetracycline and sulphametoxazole/trimethoprim. Dionisi et al. observed that *S*. Infantis ESBL-producing strains isolated from humans in Italy belonged to profiles XbaI0125, XbaI0126, XbaI0129 and XbaI2116, different from the one we found [31]. However, these profiles had the same resistance core detected by us in the profile XbaI.2621, except the resistance to cefepime. Interestingly most of the strains (93.5%) belonged to XbaI.0126 and 55% of strains having a genetic homology with the XbaI.0126 profile were ESBL producers and showed the same antimicrobial-resistance core (cefotaxime, cefepime, nalidixic acid, trimethoprim/sulphametoxazole and tetracycline). It should be underlined that *S*. Infantis ESBL isolates and cefepime-resistant strains were distributed in all pulsotypes, representing an alarming problem in both human and veterinary medicine.

## 4. Materials and Methods

### 4.1. Collection and Identification of Isolates

A total of 87 *S.* Infantis strains, isolated in Italy (North, Central and South) from 2016 to 2019, through standard ISO procedures [32], were collected in farms from fecal samples (*n* = 13) and at slaughter from environmental swabs (*n* = 11), skin and liver samples (*n* = 36) and from derived meat products (*n* = 25). The regions of Italy and the specific sources of sampling are listed in Supplementary Materials. Two human isolates were also supplied by hospitalized patients.

*Salmonella* spp. isolates were serotyped by direct slide agglutination using specific antisera (Statens Serum Institut, Copenhagen, Denmark), according to the Kaufmann-White-Le Minor scheme [33].

### 4.2. Antimicrobial Susceptibility Testing and Detection of ESBL

To assess the antimicrobial susceptibility, all *S.* Infantis isolates were analyzed on Mueller–Hinton agar plates (Thermo Fisher Scientific, MI, Italy), containing ampicillin (AMP 10 μg), amoxicillin/clavulanic acid (AMC 30 μg), ampicillin/sulbactam (AMS 20 μg), ceftazidime (CAZ 30μg), ciprofloxacin (CIP 5 μg), cefalexin (CL 30 μg), cloramphenicol (CLOR 30 μg), cefotaxime (CTX 30 μg), cefepime (FEP 30 μg), cefoxitin (FOX 30 μg), gentamicin (GENT 10 μg), imipenem (IMP 10 μg), meropenem (MEM 10 μg), nalidixic acid (NA 30 μg), trimethoprim/sulphametoxazole (SUL 25 μg) and tetracycline (TE 30 μg). The plates were incubated at 37 °C for 24 h under aerobic conditions. The results were interpreted using Clinical and Laboratory Standard Institute (CLSI) clinical breakpoints [34] with the exception of cefalexin, for which breakpoints published by EUCAST were applied [35]. Isolates showing resistance to three or more antimicrobial classes were defined as MDR [36].

ESBL production was confirmed by the combined disk test with cefotaxime and ceftazidime alone and in combination with clavulanic acid and by the microdilution method using Sensititre™ extended spectrum β-lactamase plates (Thermo Fisher Scientific, MI, Italy).

### 4.3. Characterization of ESBL-Encoding and AmpC β-Lactamase Genes

Genomic DNA was extracted from colonies using the GenElute™ Bacterial Genomic DNA Kit (Sigma-Aldrich, Darmstadt, Germany) according to the manufacturer’s protocol. The samples were analyzed spectrophotometrically and by electrophoresis on 1% agarose gel to determine respectively quantity and quality of the extracted DNA. PCR amplification of *bla_CTX-M1_* and *bla_CMY-2_* genes was performed as previously reported [37,38]. Gel electrophoresis was used for the analysis of PCR products using 2% agarose gel and ethidium bromide (0.5 μg/mL) staining.

### 4.4. Pulsed Field Gel Electrophoresis (PFGE) Analyses

Clonal relationships among the strains were assessed using PFGE according to the PulseNetprotocol. Genomic DNA was digested with XbaI (New England Biolabs, Ipswich, MA, USA), and *Salmonella enterica* subspecies *enterica* serovar Braenderup H9812 DNA was used as the molecular size marker. The gel was run on a CHEF-DRIII system (Bio-Rad Laboratories, Hercules, Ca.) over 22 h at 14 °C with 2.2–63.8 s of linear ramping at 6 V/cm [39]. Dendrogram and cluster analyses were performed using algorithms included in the BioNumerics software package v.6.6 (Applied Maths, Sint-Martens-Latem, Belgium). The percentage similarity among different chromosomal fingerprints was scored using the Dice coefficient. The unweighted pair group method with arithmetic means (UPGMA) with a 1.00% tolerance limit and 1.00% optimization was used to generate the dendrogram.

### 4.5. Statistical Analysis

Descriptive statistics were used to summarize data while distributions within categorical variables regardless of the year and matrix were analyzed using chi-square goodness of fit (each assuming all categories are equal) [40]. Resistances against antimicrobials were categorized as either resistant (=1) or sensitive (=0). Then, binomial logit models were built to assess changes in the proportion of samples that were antibiotic-resistant by year and differences according to the matrix. The human samples were excluded. The geographical area (classified as North, Central and South Italy) was excluded from the model because it was always not significant. The variables of multiresistance patterns and profiles, including ≥10 EPV were coded 0 (negative) and 1 (positive), and analyzed by using binomial logit models [41]. Estimated marginal means with standard errors, an adjusted odds ratio (aOR) with a 95% confidence interval (CI) and a *p* value were reported. The agreement between *bla_CTX-M1_*, ESBL and phenotypic resistance was evaluated by Cohen’s Kappa coefficient of agreement κ [42]. A *p*-value < 0.05 was considered statistically significant. All analyses were performed using SPSS version 25.0 statistical analysis software (IBM Inc., Chicago, IL, USA).

## 5. Conclusions

In this work, we confirmed the diffusion and persistence of the ESBL strains of *S*. Infantis, characterized by a similar multiresistance pattern, and the presence of all pulsotypes, in the meat chicken chain production, independently on the source of samples. We failed in founding correlations between multiresistance and genetic profiles. Of big concern for public health is the resistance to cephalosporines, since they are considered one of the last choices in human medicine. There is no doubt that in the fight against antibiotic-resistance it should be crucial an optimization of biosecurity plans in farming and a prudent and rational use of antibiotics in food-producing animals and in humans. Finally, it is also evident the need for longitudinal monitoring of genetic profiles addressed to better knowledge of antibiotic resistance epidemiology by applying easy-to-perform procedures, such as PGFE. A standardization of the ECDC nomenclature is therefore required in order to compare *S*. Infantis strains isolated from different geographical areas and to speculate about their circulation among various animal species, including man.

## Figures and Tables

**Table 1:** Antimicrobial susceptibility of 87 *Salmonella* Infantis isolates.

Antimicrobials	Number of Isolates (%)		
	**R**	**I**	**S**
Ampicillin-AMP	73 (84)	0 (-)	14 (16)
Cefalexin-CL	66 (76)	0 (-)	21 (24)
Cefoxitin-FOX	0 (-)	22 (25)	65 (75)
Cefotaxime-CTX	70 (80.5)	0 (-)	17 (19.5)
Ceftazidime-CAZ	10 (11.5)	30 (34.5)	47(54)
Cefepime-FEP	63 (72.4)	12 (13.8)	12 (13.8)
Nalidixic acid-NA	85 (97.7)	2 (2.3)	0 (-)
Ciprofloxacin-CIP	15 (18)	49 (56.3)	23(26.4)
Tetracycline-TE	84 (96.5)	3 (3.5)	0 (-)
Trimethoprim/sulphametoxazole-SUL	79 (91)	1 (1)	7 (8)
Cloramphenicols-CLOR	18 (20.7)	20 (23)	49 (56.3)
Gentamicin-GENT	14 (16)	0 (-)	73 (84)
Imipenem-IMP	0 (-)	9 (10.3)	78 (89.7)
Meropenem-MEM	5 (5.7)	10 (11.5)	72 (82.8)
Ampicillin/sulbactam-AMS	39 (44.8)	18 (20.7)	30 (34.5)
Amoxicillin/clavulanic acid-AMC	14 (16)	15 (17.3)	58 (66.7)

**Table 2:** Antimicrobial-resistance patterns and PCR results of 87 *S.* Infantis isolates.

No of Resistance Antibiotic Classes	Antimicrobial Resistance Pattern	N° of Isolates	% of Isolates	*bla_CTX-M1_*	*bla_CMY-2_*
**1**	beta	1	1.1%	0	0
**2**	quin/tetra	1	1.1%	0	0
**3**	beta/quin/tetra	3	3.4%	2	2
	quin/sulph/tetra	6	6.9%	0	0
	amino/sulph/tetra	1	1.1%	0	0
**4**	beta/amino/quin/tetra	4	4.6%	3	0
	beta/quin/sulph/tetra	45	51.7%	40	0
	beta/clor/sulph/tetra beta/quin/clor/sulph	1 1	1.1% 1.1%	1 1	0
**5**	beta/amino/quin/sulph/tetra	6	6.9%	6	0
	beta/carba/quin/sulph/tetra	5	5.8%	4	0
	beta/quin/clor/sulph/tetra	8	9.2%	8	0
**6**	beta/amino/carba/quin/sulph/tetra	1	1.1%	1	0
	beta/amino/quin/clor/sulph/tetra	4	4.6%	4	0

Beta-lactams(beta):ampicillin, amoxicillin/clavulanic acid, ampicillin/sulbactam, ceftazidime, cefalexin, cefotaxime, cefepime, cefoxitin; quinonlones (quin): ciprofloxacin and nalidixic acid. Tetracyclines(tetra), tetracyclines sulphonamides (sulph), trimethoprim/sulphametoxazole. Aminoglycosides (amino): gentamicin. Carbapenems (carba): imipenem and meropenem. Cloramphenicols (clor).

**Table 3:** Antimicrobial-resistance pattern of non-ESBL *S*. Infantis strains.

No of Resistance Antibiotic Classes	Antimicrobial Resistance Pattern	N° of Isolates	% of Isolates
**1**	beta	1	5.9%
**2**	quin/tetra	1	5.9%
**3**	beta/quin/tetra	1	5.9%
	quin/sulph/tetra	8	47%
	amino/sulph/tetra	1	5.9%
	amino/quin/tetra	1	5.9%
	beta/quin/sulph/tetra	3	17.6%
	carba/quin/sulph/tetra	1	5.9%

## Supplementary Materials

The following are available online at https://www.mdpi.com/2079-6382/9/11/814/s1, Material and methods S1: Sources and origin of *S*. Infantis isolates, Table S1: Resistance according to years (%) and result of pairwise comparisons, Table S2: Resistance according to matrix (%) and result of pairwise comparisons.
