# Supplementary material for: Genetic Profiles and Antimicrobial Resistance Patterns of Salmonella Infantis Strains Isolated in Italy in the Food Chain of Broiler Meat Production
Source: Antibiotics (Basel). 2020 Nov 16;9(11):814. doi: 10.3390/antibiotics9110814 (PMC7696838; doi:10.3390/antibiotics9110814)
Supplement: Supplementary file 1 [file antibiotics-09-00814-s001.pdf]

Materials and methods S1

Origin and sources of S. Infantis isolates

North Italy includes Emilia region; Central Italy includes Umbria, Marche, and Lazio regions; South Italy includes Abruzzo, Molise and Sicilia regions. The sources of sampling are classified as follows: Farm, includes faeces and litter; Environmental swabs at slaughter include conveyor belt swab, floor slaughter swab, conveyor belt swab meat skewer, conveyor belt swab breast, conveyor belt swab drumstick, conveyor belt swab meat skewer; Samples at slaughter include neck skin, chicken liver and caecal content; Derived meat products include chickens wings, minced meat, breast, thigh, meat rolle, sliced chicken breast, chicken drumstick, meat skewer, chicken drumstick, grilled chicken, portioned chicken and chicken sausage.

Table S1. Resistance according to years (%) and result of pairwise comparisons.

Antimicrobials	Years				Sig. of overall test*
	2016	2017	2018	2019	
**AMP**	100.0a	100.0a	47.4b	87.5a	***p* < 0.001**
**AMC**	26.7	7.4	0.0	29.2	*p* = 1.000
**AMS**	20.0a	48.1ab	31.6a	70.8b	***p* = 0.002**
**CAZ**	0.0	33.3	5.3	0.0	*p* = 1.000
**CIP**	46.7a	14.8ab	15.8ab	4.2b	***p* = 0.033**
**CL**	100.0a	92.6a	47.4b	70.8ab	***p* < 0.001**
**CLOR**	40.0	3.7	0.0	45.8	*p* = 1.000
**CTX**	100.0a	92.6a	47.4b	83.3a	***p* < 0.001**
**FEP**	86.7	85.2	63.2	75.0	*p* = 0.252
**FOX**	6.7	7.4	5.3	0.0	*p* = 1.000
**GENT**	6.7	3.7	0.0	50.0	*p* = 1.000
**IMP**	0.0	0.0	5.3	0.0	*p* = 1.000
**MEM**	0.0	11.1	21.1	0.0	*p* = 1.000
**NA**	100.0	100.0	94.7	95.8	*p* = 1.000
**SUL**	93.3	96.3	68.4	95.8	*p* = 1.000
**TE**	100.0	100.0	94.7	91.7	*p* = 1.000

* Overall Test results for Time effect of Models including Matrix and Time (year) effects, *p* value from Wald Chi-square. Resistances were categorized in a binary variable as resistant (=1) or sensitive (=0, including sensitive and intermediate). Values in bold are statistically significant at an alpha of 0.05.

Table S2. Resistance according to matrix (%) and result of pairwise comparisons.

Antimicrobials	Matrices				Sig. of overall test*
	Farm	Slaughter	Derived Meat Products	Environmental samples	
**AMP**	86.7	88.9	80.0	77.8	$p = 1.000$
**AMC**	26.7	19.4	8.0	0.0	$p = 1.000$
**AMS**	46.7	52.8	32.0	55.6	$p = 0.280$
**CAZ**	20.0	0.0	24.0	11.1	$p = 1.000$
**CIP**	33.3	16.7	12.0	11.1	$p = 0.603$
**CL**	86.7	80.6	76.0	55.6	$p = 1.000$
**CLOR**	20.0	41.7	0.0	0.0	$p = 1.000$
**CTX**	93.3	83.3	76.0	66.7	$p = 1.000$
**FEP**	86.7	75.0	80.0	66.7	$p = 0.715$
**FOX**	13.3	2.8	0.0	11.1	$p = 1.000$
**GENT**	6.7	36.1	0.0	0.0	$p = 1.000$
**IMP**	6.7	0.0	0.0	0.0	$p = 1.000$
**MEM**	26.7	0.0	0.0	33.3	$p = 1.000$
**NA**	93.3	100.0	100.0	88.9	$p = 1.000$
**SUL**	100.0	91.7	84.0	77.8	$p = 0.126$
**TE**	100.0	94.4	100.0	88.9	$p = 1.000$

* Overall Test results for Time effect of Models including Matrix and Time (year) effects, *p* value from Wald Chi-square. Resistances were categorized in a binary variable as resistant (=1) or not resistant (=0, including sensitive and intermediate). Values in bold are statistically significant at an alpha of 0.05.
